# Radical ligand transfer: a general strategy for radical functionalization

**DOI:** 10.3762/bjoc.19.90

**Published:** 2023-08-15

**Authors:** David T Nemoto, Kang-Jie Bian, Shih-Chieh Kao, Julian G West

**Affiliations:** 1 Department of Chemistry, Rice University, 6100 Main St MS 602, Houston, TX 77005, USA

**Keywords:** catalysis, cooperative catalysis, earth abundant elements, photocatalysis, radicals

## Abstract

The place of alkyl radicals in organic chemistry has changed markedly over the last several decades, evolving from challenging-to-generate “uncontrollable” species prone to side reactions to versatile reactive intermediates enabling construction of myriad C–C and C–X bonds. This maturation of free radical chemistry has been enabled by several advances, including the proliferation of efficient radical generation methods, such as hydrogen atom transfer (HAT), alkene addition, and decarboxylation. At least as important has been innovation in radical functionalization methods, including radical–polar crossover (RPC), enabling these intermediates to be engaged in productive and efficient bond-forming steps. However, direct engagement of alkyl radicals remains challenging. Among these functionalization approaches, a bio-inspired mechanistic paradigm known as radical ligand transfer (RLT) has emerged as a particularly promising and versatile means of forming new bonds catalytically to alkyl radicals. This development has been driven by several key features of RLT catalysis, including the ability to form diverse bonds (including C–X, C–N, and C–S), the use of simple earth abundant element catalysts, and the intrinsic compatibility of this approach with varied radical generation methods, including HAT, radical addition, and decarboxylation. Here, we provide an overview of the evolution of RLT catalysis from initial studies to recent advances and provide a conceptual framework we hope will inspire and enable future work using this versatile elementary step.

## Introduction

The behavior of alkyl radicals has been studied rigorously for decades, though only recently have these come to be widely viewed as selective and useful synthetic intermediates [1–4]. This sea change has been driven by innovations in both the generation and functionalization of alkyl radicals, with successful synthetic reactions requiring efficiency and selectivity in both of these processes and inherent compatibility between each. Radical generation has benefitted from many general mechanistic approaches, including hydrogen atom transfer (HAT) [5], alkene addition [6], and decarboxylation [7], enabling these intermediates to be easily accessed from diverse starting materials. Functionalization methods have also seen significant development, with elementary steps such as radical–polar crossover (RPC) finding significant purchase [8]; however, these steps are not amenable to all radical generation approaches/substrate classes nor can they form all desired bonds from alkyl radical intermediates, limiting the toolkit of radical reactions.

**Scheme 1:**
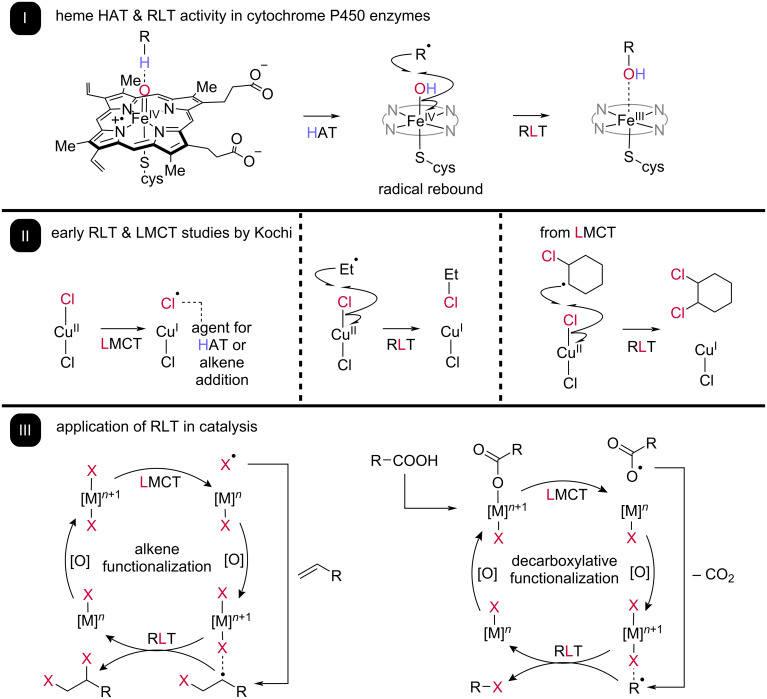
Overview of the RLT mechanism in nature and in literature. I: The radical rebound mechanism in cytochrome P450 enzymes consists of HAT on a C*–*H bond, followed by RLT with a hydroxy ligand. II: Kochi reported the oxidation of alkyl radicals through LMCT of copper(II) chloride and subsequent radical chlorine ligand transfer [26]. 1-Cyclohexene was also reported to be oxidized to the vicinal dichlorinated product through a similar mechanism. III: RLT has become more prevalent in reported catalytic cycles involving radical-based transformations including alkene difunctionalization and decarboxylative functionalization. Many of these transformations have also utilized LMCT as a means of generating reactive radical species.

Recently, radical ligand transfer (RLT) [9–11] has emerged as a radical functionalization paradigm with the potential to overcome the challenges faced by other strategies (Scheme 1). At its core, RLT involves the outer sphere transfer of an anionic, X-type ligand coordinated to a redox-active metal to a radical intermediate, resulting in formation of a new C–ligand bond with concomitant single electron reduction of the metal center. Subsequent reoxidation of the metal with coordination of a new equivalent of anionic ligand allows for the RLT complex to be regenerated, making this strategy inherently compatible with catalysis.

Building on this, one of the most important examples of catalytic RLT can be found in the human body’s own cytochrome P450 enzymes. These catalysts exhibit unique “radical rebound” reactivity at their heme active sites (Scheme 1) [12], a mechanism proposed by Groves and co-workers and heavily explored beginning in the 1970s [13–14]. This two-step functionalization sequence begins with HAT from an alkyl C–H bond to a high valent iron oxo species, resulting in formation of iron hydroxo and alkyl radical intermediates [15]. Subsequent RLT of the hydroxo ligand to the alkyl radical produces a hydroxylated product, allowing for metabolism and excretion of previously diverse bioactive compounds. Similar RLT “rebound” steps have been implicated in non-heme oxygenase and halogenase enzymes as well [16–19], hinting that this strategy might be general; however, enzymatic examples outside of hydroxo and halide ligand transfer are scarce.

**Scheme 2:**
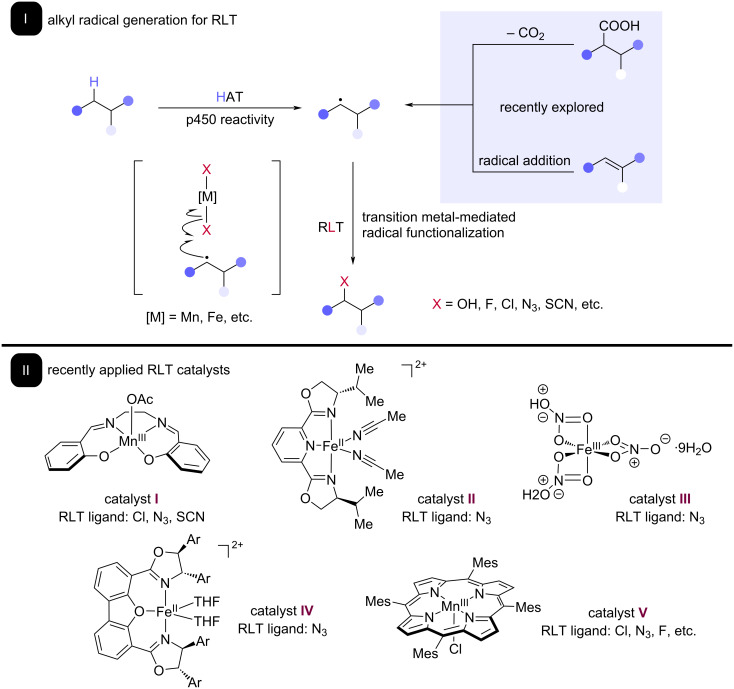
Areas of recent work on RLT development and application in catalysis. I: Reported RLT pathways often involve the generation of alkyl radicals from selective HAT on C–H bonds and, more recently, radial decarboxylation and radical addition onto π systems. Generated alkyl radicals are simultaneously quenched and functionalized through RLT. II: Modern catalysts developed for RLT catalysis.

Groves’ initial discovery of the radical rebound behavior of P450 oxygenases encouraged early work on site-selective C–H functionalization [20]. Throughout their studies, it was found that manganese could perform the same HAT and RLT steps as iron at heme active sites. Groves developed the manganese tetramesitylporphine catalyst **V** (Scheme 2), which was found to be capable of functionalizing specific C–H bonds to numerous functionalities, including C–F [21–22], C–N_3_ [23], and C–Cl bonds [24–25]. Upon these remarkable observations, methodologies involving manganese–porphyrin catalysts have been developed over the years. These methods take advantage of the power of RLT to install a variety of medicinally relevant groups, largely mirroring the selectivity of CYP450s. Intriguingly, studies by Groves have revealed earth abundant iron and manganese to be particularly privileged for this application of RLT, a major advantage for sustainable method development.

Outside of bioinorganic chemistry, the concept of radical ligand transfer was investigated in early work by Jay Kochi in purely synthetic systems (Scheme 1) [26–27]. Studies on the oxidation of alkyl radicals with earth abundant cupric salts uncovered the ability of simple Cu(II) chloride to form new C–Cl bonds in the presence of transient alkyl radicals, with mechanistic studies implicating homolytic abstraction of a chlorine ligand from the intermediate copper complex. Outside of the substitution products which could be generated from the RLT pathway, alkyl radicals could also undergo an elimination-like pathway to afford unsaturated C–C bonds in the presence of copper(II) sulfate, presumably via competitive RPC to the carbocation followed by E_1_ olefination.

Kochi also demonstrated that RLT can be combined with other radical generation strategies to enable new, non-biomimetic reactions to be achieved (Scheme 1), showing that photolysis of stoichiometric Cu(II) chloride in the presence of unactivated alkenes allows for direct formation of vicinal dichloride products. The mechanistic study implicated initial formation of a chlorine radical through homolysis of a Cu–Cl bond via ligand-to-metal charge transfer (LMCT) which, following cage escape, could add to the alkene to generate an alkyl radical. This alkyl radical could then be chlorinated via RLT from a second Cu(II) chloride species, furnishing the dichlorinated product. While copper was unable to be used catalytically in this early report, it augured the potential of RLT to be a general strategy in synthetic method development, with modern examples including new alkene addition reactions and decarboxylative functionalizations (Scheme 2).

## Recent applications of RLT in catalysis

Upon the discovery and initial exploration of the RLT paradigm by Groves and Kochi, many groups have adopted and characterized new ways of using RLT to form valuable carbon–heteroatom bonds from a diverse pool of simple starting materials. RLT has been especially present in modern catalysis, where complexes of earth abundant iron and manganese have been demonstrated to be particularly privileged in delivery of various ligands to alkyl radicals (Scheme 2). These developments have been supported by discovery of the compatibility of RLT with many different reaction paradigms leading to alkyl radical intermediates under catalytic conditions, including radical addition to alkenes and radical decarboxylation, with many of these being driven by light energy.

### RLT in alkene functionalization

**Scheme 3:**
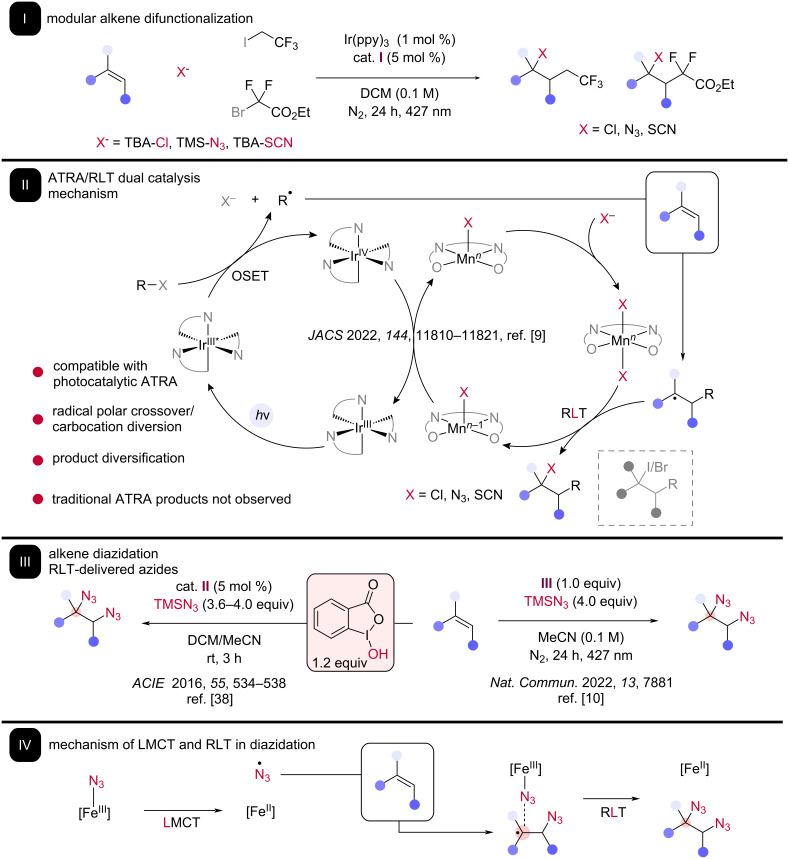
The incorporation of RLT catalysis in ATRA photocatalysis. I: The reported method is compatible with nucleophilic sources of chlorine groups, azide groups, and thioisocyanate groups. II: The proposed mechanism for the dual catalytic ATRA-RLT cycle. III: Our lab and Xu reported photochemical diazidation of alkenes carried out using iron and trimethylsilyl azide. IV: The proposed mechanism for photoinduced LMCT between iron and azide ligands as well as RLT on azidoalkyl radical intermediates.

Outside of the realm of C–H activation, RLT has been leveraged to afford complex medicinal scaffolds in alkene difunctionalization. A recent example can be found in the merger of RLT with photoredox-catalyzed atom transfer radical addition (ATRA) (Scheme 3). ATRA results in the net addition of a C–X bond across an alkene, forming both valuable C–C and C–X bonds in a single reaction. While ATRA-type reactions were first reported in the 1940s by Kharasch [28], interest in the area was revitalized the early 2010s with the advent of Stephenson’s photoredox catalytic methods which dramatically simplified reaction conditions [29–30], driving ongoing interest in this mechanistic approach [31].

Our group recently devised a dual catalytic method which combines the RLT paradigm with photocatalytic ATRA to enable the modular difunctionalization of alkenes under reagent control (Scheme 3). In Stephenson’s photocatalytic ATRA reports, the C–X bond in the product was proposed to be formed through both direct quenching of a transient alkyl radical by halogen atom transfer (XAT) from the alkyl halide reagent and further oxidation of the transient radical to a carbocation by radical polar crossover (RPC), providing two mechanistic pathways to form the ATRA products [32]. While powerful, this approach is inherently incompatible with introducing alternative functionality instead of the halide included in the alkyl halide reagent, limiting the ability to form different difunctionalization products. Taking inspiration from Groves’ bio-inspired manganese tetradentate manganese catalysts, we found we could instead functionalize the transient alkyl radical via RLT from a simplified manganese salen complex **I**, allowing for the identity of the carbon–heteroatom bond to be controlled based on added nucleophile and enabling C–Cl, C–N, and C–S bonds to be formed directly while completely suppressing traditional ATRA products [9]. In mechanistic studies, rearrangement products indicative of a carbocationic pathway are not observed, suggesting that RPC does not occur. Further, the inability of ATRA products to undergo S_N_2 with the added nucleophiles under our reaction conditions is inconsistent with a tandem ARTA/nucleophilic displacement alternative mechanism. Finally, a functionalization via the canonical organometallic steps of oxidative addition/reductive elimination was ruled out via catalytic reaction of the macrocyclic Groves-type porphyrin catalyst **V**, a species that is unable to accommodate the mutual cis-orientation of ligands for metal-centered reductive elimination. The system was found to be compatible with unactivated alkenes bearing a wide range of functionalities, including more-substituted alkenes, and a wide range of alkyl halide reagents, permitting a library of difunctionalized products to be prepared from a single alkene.

RLT can also be used to deliver valuable homodifunctionalized products using unactivated alkenes. Vicinal diazides (and to a lesser extent dihalides) have been popular targets for modern method development. Both photochemical [33] and electrochemical [34–36] methods have been effective in delivering products containing these molecular motifs. Intriguingly, several recent alkene diazidation methods have made RLT a key design criterion, with both thermal and photochemical driving forces [37].

Recent interest in alkene diazidation was accelerated by a 2016 report from Xu detailing alkene diazidation using low loadings of a molecular iron catalyst **II** and stoichiometric hydroxyiodinane as a terminal oxidant [38]. It is proposed that an azidoiodinane is generated in situ and serves as the radical initiator, generating an azido radical which adds to the less substituted position on the alkene. The resultant transient radical is captured via RLT from an in-situ generated iron–azide complex, resulting in net reduction of iron. The competent RLT species can then be regenerated through oxidation by the iodinane species and coordination of another equivalent of azide. This reaction was particularly notable for the wide alkene scope, including terminal aliphatic alkenes, internal (cyclic) styrenes, and one example of a nonconjugated diene, suggesting RLT to be compatible with many functionalities. The diastereoselectivity of the reaction varies, with high anti-selectivity being achieved with cyclic styrenes and low diastereoselectivity in linear internal alkenes.

Building on this key iron catalysis result, our group and that of Shi contemporaneously reported the photochemical diazidation of alkenes using stoichiometric iron and no external oxidant or ancillary ligand, providing a simple protocol for the preparation of vicinal diamines with excellent functional group compatibility (Scheme 3) [10,39]. In both reports, it is proposed that photoinduced LMCT of an in-situ generated Fe(III) azide species furnishes an azido radical, compatible with unactivated alkene addition. These steps provide the reactive carbon-centered radical intermediate. RLT to this radical from another azide ligand leads to a diazidated product. The overall scope of both reports suggests that the diazidation of simple to complex drugs/natural product-derived alkene substrates is readily achievable, including highly substituted and cyclic aliphatic alkenes. Further, we demonstrated that diazidation could be rendered catalytic using Fe(III) nitrate hydrate **III** as the iron source and performing the reaction under continuous flow conditions. Interestingly, this mechanism bears some similarity to Lin’s electrocatalytic diazidation, where azido radical generation is proposed via thermal homolysis of a Mn(III) azide species and RLT from a second equivalent of Mn(III) azide furnishes the desired organic diazide, providing a strong demonstration of the applicability of RLT to not only photochemical but electrochemical conditions as well [35].

### RLT in decarboxylative functionalization

Aside from its strategic application in alkene difunctionalization methods, RLT has also found synthetic utility in radical decarboxylative reactions. Radical decarboxylative functionalization reactions to form C–X bonds have been demonstrated, with bond construction being proposed to follow one of two pathways: formation of a carbocation through RPC followed by nucleophilic attack or direct RLT from a redox-active metal complex.

**Scheme 4:**
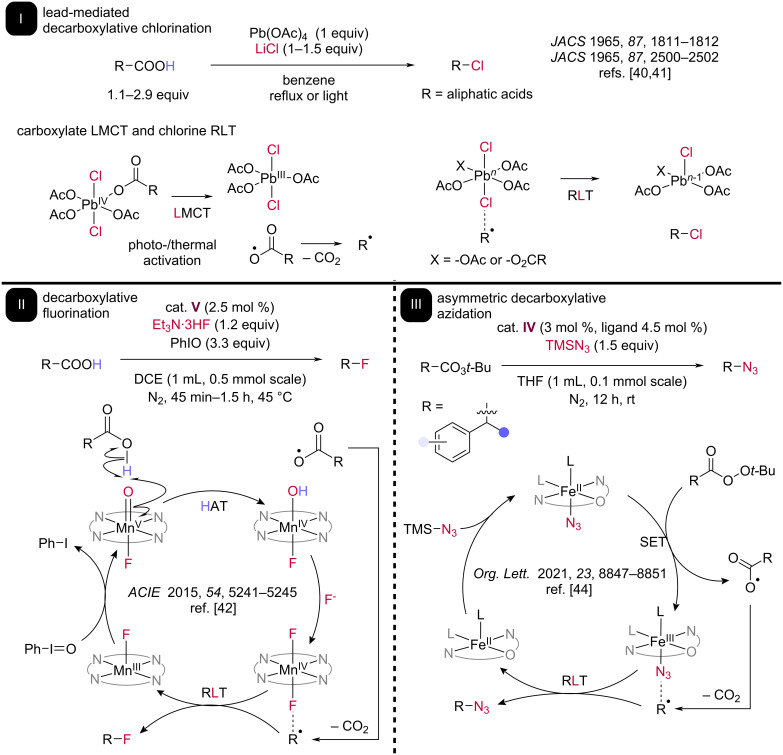
Pioneering and recent work on decarboxylative functionalization involving a posited RLT pathway. I: In 1965, Kochi reported thermal and photochemical decarboxylative chlorination using stoichiometric lead(IV) acetate. LMCT on the carboxylate substrate and RLT on a Pb–Cl bond are supported by mechanistic studies. II: Groves reported decarboxylative fluorination employing catalyst **V**. III: Bao reported asymmetric decarboxylative azidation through use of BOX-derived iron catalyst **IV**.

Preliminary evidence for a radical decarboxylation/RLT cascade was reported in 1965, when Kochi demonstrated decarboxylative chlorination of various acids with lead(IV) tetraacetate in the presence of lithium chloride (Scheme 4) [40–41]. Nucleophilic lithium chloride was used as the chlorine atom source for this transformation. In the representative scope of this transformation, primary and secondary chlorides could be formed in relatively high yields from their respective acids, a result incompatible with a carbocation RPC mechanism. This Kochi decarboxylative chlorination separated itself from other pioneering methods of decarboxylative functionalization (i.e., Barton and Hunsdiecker) because of its inclusion of RLT as a key element of the mechanism.

In 2015, the Groves group reported their manganese porphin-based catalyst **V** and related species being capable of participating in decarboxylation reactions (Scheme 4) [42]. The activated Mn(V) species is proposed to perform HAT carboxylic acid O–H bond, directly forming a carboxyl radical and Mn(IV) species which can exchange its hydroxo ligand for a fluoride from triethylamine tris(hydrofluoride) (Scheme 4). Rapid decarboxylation of this intermediate produces the alkyl radical species which could be fluorinated via RLT from the Mn(IV)–F complex, generating a Mn(III) intermediate. To close the cycle and reform the oxo ligand on the Mn(V) species, a stoichiometric amount of iodosylbenzene is used in the reaction.

While initial development of this reaction focused on incorporating the stable ^19^F, subsequent study expanded the scope to RLT of the unstable ^18^F radioisotope, an important medical radioisotope used for positron emission tomography (PET) [43]. Optimized conditions of both isotopes included fast reaction times of under two hours; in the case of the ^18^F radioisotope, reactions were carried out in 10 minutes and resulted in moderate to high yields, demonstrating the potential of RLT reactions to be rapid and efficient. In both cases, benzylic carboxylic acids were most amenable as substrates, with alkyl carboxylic acids such as adamantane and dicyclohexylmethane providing fluorinated aliphatic products in low to moderate yields.

Asymmetric RLT catalysis has also been of recent interest, with exciting preliminary decarboxylative azidation results obtained under thermal conditions by Hongli Bao and co-workers [44]. An asymmetric iron (NON) pincer catalyst **IV** was employed to decarboxylate benzylic peroxyesters and form enantiomerically enriched benzylic azides. An Fe(II/III) cycle is proposed, where a single electron transfer from Fe(II) reduces the peroxyester and produces a carboxyl radical and Fe(III), which can coordinate an azide ligand. Rapid decarboxylation produces the transient alkyl radical which can be asymmetrically azidated by RLT from an Fe(III) azide complex, reducing the iron catalyst back to the starting Fe(II) state. Organic azides can be formed in moderate to high enantioselectivity using this approach; however, the scope is largely limited to benzylic products, a result in line with Groves’ finding that benzylic acid substrates perform much more efficiently in decarboxylative RLT reactions than aliphatic acids [42]. Outside of decarboxylation, X. Peter Zhang recently reported the enantioselective synthesis of allylic amines through coupled HAT and RLT on allylic C–H bonds [45], using a bulky cobalt porphyrin complex developed and utilized to perform both HAT and RLT. While many challenges remain for achieving general asymmetric induction in RLT catalysis, this advance represents an important step toward this aspirational goal with many lessons to build upon.

**Scheme 5:**
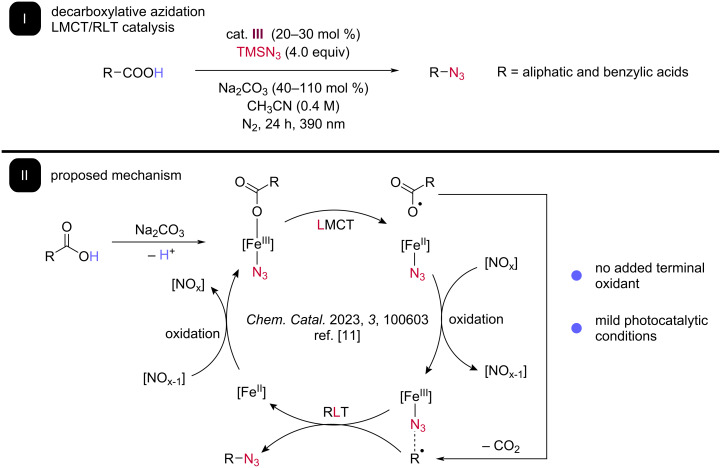
Our lab reported decarboxylative azidation of aliphatic and benzylic acids. I: The reaction proceeds via LMCT and RLT catalysis without the addition of terminal oxidant. II: The proposed mechanism includes reoxidation of the iron catalyst through inner-sphere electron transfer by anionic nitrate.

Our group has recently leveraged iron photochemistry to build on these beautiful decarboxylative azidation examples, combining iron-mediated photodecarboxylation via LMCT and azide RLT (Scheme 5) [11]. Irradiating a substoichiometric amount of iron(III) nitrate hydrate **III** in the presence of carboxylic acid, TMS azide, and sodium carbonate allows for direct synthesis of alkyl azides for a wide range of both activated (benzylic) and unactivated carboxylic acids. Control reactions support the intermediacy of alkyl radicals and the absence of carbocation rearrangements in a variety of probe substrates disfavor the reaction proceeding via RPC.

Intriguingly, no additional oxidant is required for this process, implicating the nitrate counterion functioning as an internal oxidant to regenerate the active Fe(III) species capable of LMCT and RLT. This result is consistent with our finding that iron nitrate can catalytically diazidate alkenes in flow with no additional oxidant and literature examples of nitrate oxidation of different transition metals, such as palladium. Control reactions further supported this proposal, including the inability of alternative Fe(III) salts (e.g., FeCl_3_) to form more than stoichiometric azide product in the absence of added nitrate. We believe this adventitious discovery of nitrate functioning as a mild and selective oxidant in RLT catalytic systems presents many opportunities for future method development and are avidly pursuing this area of research.

## Outlook

After scant exploration following its elucidation in early mechanistic studies of bioinorganic and synthetic systems, radical ligand transfer (RLT) has reemerged as a powerful tool in the design of catalytic radical reactions. This development has been fueled by the unique aspects of RLT, with its ability to functionalize radicals with diverse nucleophilic reagents and inherent compatibility with different elementary steps, including hydrogen atom transfer (HAT) and ligand-to-metal charge transfer (LMCT), enabling new transformations to be unlocked with unprecedented modularity. Further, the privileged position of earth abundant elements such as iron and manganese in RLT has made reactions using this step appealing from a sustainability standpoint. While exciting progress has been made, many opportunities remain using this mechanistic approach. Two key areas that could yield exciting advances are combining RLT with new radical-generating elementary steps and the further development of asymmetric RLT processes. We hope that this perspective provides a useful framework for understanding RLT reactivity and inspires new advances using this versatile and intriguing elementary step.
